# Hypoactive Delirium: A Rare Manifestation of Scrub Typhus

**DOI:** 10.7759/cureus.70740

**Published:** 2024-10-02

**Authors:** Asif Dabeer Jafri, Srikant K Dhar, Chitralekha Naik, Kayenaat Rizvi

**Affiliations:** 1 General Medicine, SUM Ultimate Medicare, Bhubaneswar, IND; 2 Internal Medicine, Institute of Medical Sciences (IMS) and SUM Hospital, Bhubaneswar, IND; 3 Neurology, Sum Ultimate Medicare, Bhubaneswar, IND; 4 Pharmacology, Era's Lucknow Medical College and Hospital, Lucknow, IND

**Keywords:** hypoactive delirium, modafinil in scrub typhus, neuroimaging in scrub tyhus, neuropsychiatric symptoms in scrub typhus, scrub typhus treatment

## Abstract

Scrub typhus continues to pose a significant threat to life, manifesting in a spectrum that ranges from mild, non-specific febrile illness to severe multi-organ dysfunction. Although neuropsychiatric symptoms are rare in cases of scrub typhus, we present a unique case involving a 60-year-old male who initially exhibited fever and headache, subsequently developing neuropsychiatric symptoms on the third day of hospitalization. Following the exclusion of prevalent metabolic, autoimmune, and infectious conditions, he was diagnosed with hypoactive delirium associated with scrub typhus. This case highlights the complex nature of hypoactive delirium, which may manifest with nonspecific symptoms that are frequently overlooked. Consequently, the recognition of delirium can be particularly difficult, potentially resulting in underdiagnosis in clinical settings.

## Introduction

Scrub typhus is an acute infection caused by the rickettsial organism *Orientia tsutsugamushi*, which is transmitted through the bite of an infected mite [1]. The neurological involvement of scrub typhus includes both the central and peripheral nervous systems [2,3]. The neurological symptoms associated with scrub typhus are relatively frequent and exhibit considerable diversity. Meningoencephalitis is a well-recognized manifestation of scrub typhus; other reported neurological complications include cranial nerve palsies, plexopathy, transverse myelitis, cerebral venous thrombosis, cerebrovascular accident, parkinsonism, cerebellitis, and Guillain-Barré syndrome. However, neuropsychiatric manifestations are rare in scrub typhus and are reported as depression, hallucination, post-infectious obsessive-compulsive disorder, and delirium [4]. Delirium is a severe form of acute confusional state that is marked by mental instability, altered consciousness, and inattentiveness. Delirium is classified into two distinct types: hypoactive and hyperactive, which are determined by the degree of alertness and motor activity. Hypoactive delirium is often characterized by lethargy and apathy, whereas hyperactive delirium is marked by restlessness and hostility [5]. The exact pathophysiological mechanisms of scrub typhus-associated psychiatric manifestations remain unclear; oxidative stress, endotheliopathy, and increased vascular permeability may play significant roles. Pathological findings indicative of central nervous system involvement in scrub typhus include the presence of microglial cell clusters, diffuse or focal infiltration of mononuclear cells in the leptomeninges, and abnormalities in the white matter, particularly affecting the subcortical, periventricular, and deep white matter regions.

Several modalities may aid in diagnosis, including enzyme-linked immunosorbent assay (ELISA), immunofluorescence assay (IFA), immunochromatographic test (ICT), Weil-Felix, polymerase chain reaction (PCR), and loop-mediated isothermal amplification (LAMP). Though frequently used in the past, the Weil-Felix test is no longer preferred due to its limited accuracy in both specificity and sensitivity [6]. Examination of the cerebrospinal fluid (CSF) may detect elevated protein levels and lymphocytic pleocytosis [7]. Deranged liver enzymes, anaemia, leukocytosis, thrombocytopenia, hypoproteinaemia, and impaired renal function were among the nonspecific findings of routine laboratory tests. The neuroimaging characteristics of scrub typhus in encephalitis are not extensively recognized [8]. Furthermore, Sood et al. have indicated that advanced magnetic resonance imaging (MRI) techniques can significantly enhance the differentiation between grey and white matter, allowing for the detection of brain abnormalities that may not be apparent on conventional MRI scans [9]. Malaria, dengue fever, leptospirosis, and chikungunya should be considered as differential diagnoses for patients exhibiting altered mental status alongside laboratory findings indicative of thrombocytopenia, coagulopathy, and impaired liver or kidney function. While the majority of scrub typhus cases are moderate and self-resolving, a minority can present with prolonged and severe symptoms, potentially leading to fatal outcomes if appropriate antibiotic treatment is not initiated on time. The drug of choice is doxycycline [10]. Other antibiotics for the treatment of scrub typhus include azithromycin, chloramphenicol, and rifampicin. In the treatment of hyperactive delirium, antipsychotics have been extensively studied. Other than addressing delirium's predisposing and precipitating conditions, such as infection, polypharmacy, or electrolyte and fluid imbalances, there is little agreement on how to treat hypoactive delirium.

## Case presentation

A 60-year-old male with no significant past medical history presented to our hospital with a history of fever, headache, and arthralgia for seven days. Despite regular administration of acetaminophen, his fever remained unabated. Upon admission, he was alert and oriented, with stable hemodynamics. Diagnostic tests confirmed a positive result for scrub typhus, leading to the initiation of oral doxycycline treatment. Following the commencement of therapy, the patient exhibited gradual improvement, became afebrile, and was prepared for discharge. However, on the day of discharge, he suddenly developed mutism, showing reluctance to eat and refraining from verbal communication, although he remained conscious. On physical examination, he was drowsy but easily arousable, with no orientation to time, place, or person. His vitals at the time remained stable (heart rate 84/min, respiratory rate 20/min, blood pressure 120/80 mmHg, and oxygen saturation of 98% on room air). Evaluation of fluid status indicated euvolemia. There was no eschar, no signs of meningismus, and the rest of the neurological examination (limited by participation) was unremarkable. IV (intravenous) acyclovir and IV dexamethasone were added, and oral doxycycline was switched to IV doxycycline after discussion with the neurologist. The patient's condition continued to be the same; hence, steroids and acyclovir were stopped on day 7. With no improvement, a repeat lumbar puncture was performed, which showed a normal study. A psychiatrist consultation was requested, and he advised antipsychotics and benzodiazepines (sertraline and lorazepam), which were also stopped after six days as no significant improvement was noticed. A repeat electroencephalogram (EEG) was performed, which showed the same changes as before. As there was no improvement observed, we discontinued the use of doxycycline on the 16th day. We initiated treatment with modafinil after discussing it with a neurologist, which resulted in a remarkable transformation. Within just two doses of modafinil, he exhibited significant improvement in his sensorium, becoming alert, talkative, and even walking in the ward corridor. He also started to talk to his relatives, answered phone calls, and became aware of his bowel and bladder movements, proving a dramatic change in his condition. Laboratory parameters of our patient are shown in Table 1.

**Table 1:** Laboratory parameters at admission S. Na: Serum sodium; S. K: Serum potassium; SGOT: Serum glutamic oxaloacetic transaminase; SGPT: Serum glutamate pyruvate transaminase; TSH: Thyroid stimulating antibody

Analyte	Patient result	Reference value
Haemoglobin	11.9	13-17 g/dL
Total leucocyte count	9.42	4-11 x 10^9/L
Total platelets count	351	150-400 x 10^9/L
S. Na	137	135-145 mEq/L
S. K	3.8	3.5-5 mEq/L
SGOT	123.5	5-40 U/L
SGPT	127.1	5-40 U/L
Total bilirubin	0.89	0.0-2.0 mg/dL
Alkaline phosphatase	106	40-129 IU/L
Total protein	6.49	6.0-8.3 g/dL
Serum albumin	4.29	3.3-5.2 mg/dL
Urea	20	13-45 mg/dL
Creatinine	0.8	0.5-1.5 mg/dL
Calcium	8.4	8.6-10.3 mg/dL
TSH	1.90	0.270-4.20 mIU/L

**Figure 1:**
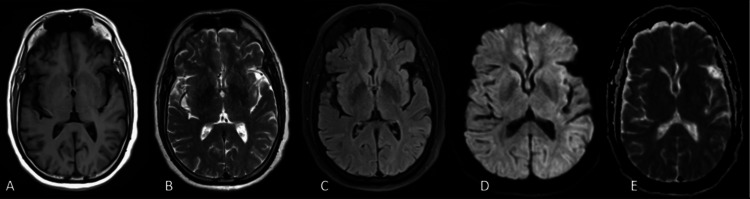
(A) T1-weighted imaging; (B) T2-weighted imaging; (C) T2-FLAIR imaging; (D) DWI; (E) Corresponding ADC map shows no significant abnormality FLAIR: Fluid-attenuated inversion recovery; DWI: Diffusion-weighted imaging; ADC: Apparent diffusion coefficient

The serological tests for dengue, leptospira, malaria, and salmonella returned negative. Additionally, tests for HIV, as well as hepatitis A, B, C, and E, were also negative. The contrast-enhanced MRI (CEMRI) brain scan was normal, as shown in Figure 1.

**Figure 2:**
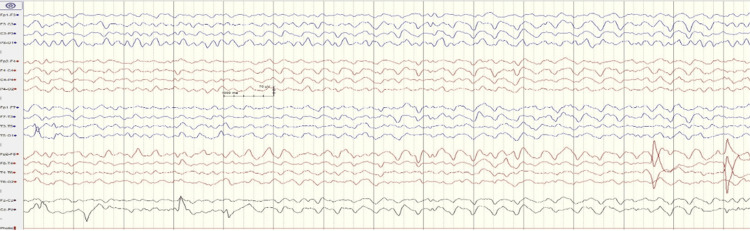
EEG shows diffuse slow waves in the range of theta (4-7 Hz) activities, intermixed with delta waves No paroxysmal sharp or spike-wave discharges are noted EEG: Electroencephalogram

Furthermore, the CSF analysis showed no abnormalities. All cultures, encompassing blood, CSF, and urine, yielded sterile results. CSF viral panels were also negative. The EEG displayed diffuse slow wave activity within the theta range (4-7 Hz), interspersed with delta waves, indicating an encephalopathic pattern, as illustrated in Figure 2.

The patient was initially given oral doxycycline, but on the third day, when his sensorium was altered, oral doxycycline was switched to IV doxycycline. IV acyclovir and IV dexamethasone were added, which were stopped after seven days, as there was no improvement in his condition. Antipsychotics and benzodiazepines (sertraline and lorazepam) were added the next day after the stoppage of steroids and were given for six days, which were subsequently stopped as no improvement was noticed. Modafinil 100 mg twice daily was added, which showed a drastic change. After an 18-day inpatient stay, he was discharged on modafinil, 50 mg twice daily. During his follow-up appointment, all medications were discontinued. The patient continues to receive regular follow-up care and is reported to be in good health.

## Discussion

Neuropsychiatric manifestations in scrub typhus are not frequently described in the literature. Brain imaging can be normal in patients with scrub typhus-associated neurological abnormalities [11]. The pathophysiology underlying the psychiatric manifestations involves the direct invasion of the causative organism into the central nervous system. The bacteria can infiltrate the endothelial cells of blood vessels, leading to an inflammatory response. This inflammatory cascade initiates the release of cytokines and other immune mediators, contributing to neuroinflammation. These pathophysiological processes can result in various psychiatric manifestations, including delirium, psychosis, mood disorders, and cognitive impairment. The severity and specific nature of psychiatric symptoms can vary among individuals, and prompt recognition and appropriate management are crucial for optimal patient outcomes.

It has been shown in a recent nationwide analysis that patients with tsutsugamushi infection have a 1.56-fold higher risk of depression than the general population [12]. Ripley has noted that neuropsychiatric manifestations may occur in individuals with scrub typhus [13]. The analysis of cerebrospinal fluid appears normal, and the lack of response to doxycycline, coupled with a rapid response to modafinil, suggests the exclusion of encephalitis and indicates a neuropsychiatric manifestation associated with scrub typhus. Hence, hypoactive delirium should be included in the differential diagnosis for altered sensorium associated with scrub typhus in endemic regions. Timely identification and management of this condition can facilitate the early discharge of the patient and avoid unnecessary escalation of antibiotics. More research needs to be conducted to better understand stimulants' role in individuals with hypoactive delirium, but the evidence at present is insufficient to advocate their routine use.

## Conclusions

Clinicians should consider the possibility of scrub typhus-associated neuropsychiatric manifestations in patients presenting with fever and altered sensorium, particularly in endemic areas. The absence of eschar and the unremarkable results from neuroimaging could potentially result in a scenario where this otherwise manageable condition leads to prolonged hospitalization if not identified on time.
